# Effect of light-emitting diode curing tip distance on shear bond strength in metal, ceramic and polycarbonate orthodontic brackets: An *in vitro* study

**DOI:** 10.6026/973206300222750

**Published:** 2026-05-31

**Authors:** Hemadri1 S, Kala Vani S.V, Firoz Babu Palagiri, Vijaysinh R Tanpure, VLakshmi Sagar, Thejaswini1 N

**Affiliations:** 1 Department of Orthodontics, CKS Teja Institute of Dental Sciences and Research, Tirupati, Andhra Pradesh, India; 2 Department of Orthodontics, Yashwantrao Chavan Dental College, Ahilyanagar, Maharashtra, India

**Keywords:** Orthodontic brackets, shear bond strength, LED curing, distance between curing tip, ceramic brackets, polycarbonate brackets

## Abstract

Adequate shear bond strength between orthodontic brackets and enamel is essential for successful treatment. However, the effect of LED curing tip distance on different bracket materials remains unclear. Therefore, it is of interest to evaluate the shear bond strength of stainless-steel, polycarbonate and monocrystalline ceramic and polycrystalline brackets at varying LED curing distances. Two hundred premolars were divided by bracket type and curing distances (0, 6, 8, 10 and 15 mm) and bond strength was tested using a universal testing machine. Results showed high bond strength at 0 mm with a significant reduction as distance increased, with stainless-steel and ceramic brackets maintaining clinically acceptable values while polycarbonate brackets showed reduced strength at greater distances. Both bracket material and curing distance significantly influenced shear bond strength, emphasizing the need to minimize curing distance for optimal bonding performance.

## Background:

The direct attachment of orthodontic brackets to the enamel surfaces is one of the most basic activities in the modern fixed appliance therapy. Since the initial introduction of the acid-etch technique that made the basis of adhesive bonding in dentistry, direct bonding has progressed to be a stable and universally implemented clinical procedure that allows efficient transmission of orthodontic force, as well as the conservation of the tooth structure[1].The clinical effectiveness of bonded orthodontic attachments hinges on the success attainment of adequate bond strength which is capable of withstanding the displacement forces that are created in the process of mastication, orthodontic mechanics and incidental contact during the course of treatment [2].The most commonly used laboratory parameter of measuring the outcomes of orthodontic bonding systems is shear bond strength. The values of bond strength that lie between 6 and 8 megapascals have been determined to be typically adequate to resist the forces that occur during the normal course of the orthodontic treatment, yet at the same time allow safe debonding without causing an iatrogenic enamel loss on completion of treatment[3, 4]. Several variables lead to the general bonding performance, among them are the enamel surface conditioning, resin composition, the shape of the base of the bracket, curing protocol and the innate material characteristics of the bracket material itself [5]. [6]. The development of the bracket materials has been motivated to a great extent by the rising aesthetic demands of the orthodontic patients. Stainless-steel brackets have remained the most widely used type of bracket , due to their outstanding mechanical traits, good bonding attributes which are due to its base in the form of a mesh and because they have been clinically reliable over time [7].However, the high visibility of metal brackets has triggered the creation of more aesthetically better alternatives. Ceramic brackets, which come in monocrystalline (sapphire) and polycrystalline (alumina) types, a re highly colour stable and optically translucent, which is almost similar to that of natural tooth structure [8, 10].

The polycarbonate brackets are another aesthetic choice to offer a decent appearance at a lower price, but the problem of low mechanical strength and easy deformation as well as bonding success has always been reported in the literature[11,12]. Extended working hours and precise polymerization initiation control are some of the benefits associated with the widespread usage of light-activated orthodontic adhesive systems by clinicians. Light-emitting diode curing systems have increasingly replaced traditional halogen-based units with the advantages of their narrower and more focused spectral output at the absorption maximum of camphorquinone photoinitiators, low energy consumption, long working life and decreased heating effect at the tooth surface [13,14]. Nevertheless, the efficiency of polymerization of LED curing devices is vulnerable to various clinical factors such as cumulative exposure, the strength of the emitted light and the angulation of the guide of the light with the bracket as well as, most importantly, the distance between the curing tip and the adhesive-bracket interface [15,17]. Curing tip distance is clinically most relevant in areas of bonding, especially in the posterior dentition where the anatomical limitations, restriction of the mouth and access of visual areas often prohibit the optimal placement of the light guide in close proximity to the bracket surface. The further the curing tip is to the target surface, the greater will be the dispersion and attenuation of light energy and consequently the lesser will be the irradiance at the adhesive layer and the possibility of full polymerization of the resin matrix [18]. Past studies have indicated the inversely proportional relationship between curing distance and the level of curing resin polymerization, indicated by low surface hardness and undermined mechanical characteristics [19,20]. Therefore, it is of interest to assess and compare the impact of the distance between LED curing tip and the shear bond strength of the stainless-steel, polycarbonate and monocrystalline ceramic and polycrystalline ceramic orthodontics brackets bonded to the human enamel with the help of a conventional light-cured adhesive system.

## Materials and Methods:

## Design and preparation:

This was an *in vitro* experimental experiment based on 200 human maxillary first premolars that had been extracted using the orthodontic procedure. The teeth were thoroughly observed under magnification and the specimens with carious lesions, restorations, enamel fractures, surface cracks and developmental defects were not included in the study. The chosen teeth were completely debrided of the remaining soft tissue and calculus deposits with hand scalers, rinsed in running water and kept in physiological saline solution at room temperature until usage. Bonding procedures were all done in three months of extraction to limit changes in the enamel surface properties.

## Grouping and allocation:

The 200 specimens were randomly divided in four main groups of 50 teeth each depending on the kind of orthodontic bracket to be bonded:

The stainless-steel brackets:

Group I: The brackets of

Group II: are polycarbonate brackets.

Group III: Monocrystalline ceramic (sapphire) brackets.

Group IV: PLCs consists of ceramic brackets.

The major groups were further divided into five subgroups of 10 specimens each, which were the five distances of the LED curing tips studied in this research, namely; 0 mm (direct contact), 6 mm, 8 mm, 10 mm and 15 mm.

## Specimen mounting:

Every tooth was mounted vertically on a self-curing block of acrylic resin in a way that the buccal side of the crown was set free and in a direction that was perpendicular to the flow of shear force imagination. The acrylic blocks were also standardized in size to make sure that they could fit in the same way in the testing apparatus.

## Bonding Procedure:

Each of the specimens was rinsed in water by immersing in a rubber prophylaxis cup and applying fluoride-free pumice slurry on its buccal enamel surface, followed by 10 seconds of rinsing in water and air drying. Enamel conditioning was conducted taking 37% phosphoric acid gel on the buccal surface and covering it with 15 seconds. The etched surface was then sprayed with water over 20 seconds then dried with compressed air, which was oil-free so that a typical frosty white surface was seen, which indicated a sufficient level of enamel etching. Orthodontics bonding primer was used to provide a thin, uniform layer on the surface of the etched enamels using a micro-brush applicator and cured under the LED curing unit over a period of 10 seconds. The correct type of bracket was then placed on the center of the buccal surface. Bracket base Light-cured orthodontic adhesive paste was then placed on the base of the bracket, the bracket was firmly seated on the enamel surface and any residual adhesive was removed carefully on the bracket margins with a dental explorer. The curing was done with one curing unit of LED with a known output of about 1000 mW/cm^2^. A total of 40 seconds were spent to cure each specimen using the corresponding curing distance. In order to equalize the curing distance, custom-made spacing devices of predetermined thickness (0, 6, 8, 10 and 15 mm) were placed between the curing tip and the buccal enamel surface. The light guide was kept at the right angle of the bracket surface during the curing cycle. After bonding, all specimens were kept in distilled water at 37°C overnight before mechanical testing so that post-cure polymerization would be complete and to render the specification as close to the actual oral environment when wet.

## Shear bond strength testing:

The testing specimens were made fixed on the testing jig of a universal testing machine in a manner that the occluso-gingival axis of the bracket base was perpendicular to the direction of force applied. A chisel-edged blade was placed at the interface between the bracket and the adhesive enamel where compressive shear was applied at a constant crosshead speed of 1mm/min until bracket debonding was possible. The highest force in the failure was given in Newtons.

The shear bond strength was determined as follows:

SBS (MPa) = Force at failure (N)/ Bracket base area (mm^2^)

## Statistical analysis:

All the data were converted into a statistical software package (SPSS version 22.0; IBM Corp., Armonk, NY). Each group and subgroup was divided into descriptive statistics comprising of means and standard deviations. ANOVA on the two parameters of bracket type and curing tip distance and the interaction effect with shear bond strength was analyzed using the two-way analysis of variance (ANOVA). The pairwise comparisons between individual groups were done using Tukey honestly significant difference (HSD) test. The statistical significance was set at p < 0.05.

## Results:

The mean shear bond strength values for all four bracket types at each curing tip distance are presented in Table 1. Across all bracket materials, the highest SBS values were consistently recorded at the 0 mm curing distance, with a progressive and measurable decline in bond strength as the curing tip distance increased to 6, 8, 10 and 15 mm. Among the bracket types, polycrystalline ceramic brackets demonstrated the highest mean SBS at most curing distances, followed closely by monocrystalline ceramic and stainless-steel brackets. Polycarbonate brackets exhibited the lowest mean SBS values at all curing distances. At the maximum curing distance of 15 mm, the mean SBS of polycarbonate brackets (4.71 MPa) fell below the widely accepted clinical threshold of 6 MPa, while stainless-steel (8.77 MPa), monocrystalline ceramic (10.23 MPa) and polycrystalline ceramic brackets (10.62 MPa) maintained values above this threshold. Two-way ANOVA revealed that both bracket type and curing tip distance exerted statistically significant main effects on shear bond strength (p < 0.001 for both factors). Furthermore, the interaction between bracket type and curing distance was also statistically significant (p<0.05), indicating that the magnitude of bond strength reduction with increasing distance varied according to bracket material (Table 2). Tukey's post-hoc analysis revealed that polycarbonate brackets demonstrated significantly lower SBS values compared to stainless-steel, monocrystalline ceramic and polycrystalline ceramic brackets at all curing distances (p < 0.001). No statistically significant difference was found between monocrystalline and polycrystalline ceramic brackets at curing distances of 0, 6, 8 and 10 mm (p > 0.05), although polycrystalline ceramic brackets showed marginally higher values. Stainless-steel brackets exhibited SBS values comparable to monocrystalline ceramic brackets at 0 and 6 mm distances but demonstrated significantly lower values than both ceramic types at distances of 10 and 15 mm (p < 0.05)(Table 3).

## Discussion:

The current research examined how the distance of the LED curing tip and the type of material used in the brackets affected the shear bond strength of orthodontic brackets bonded to the enamel of humans. The findings were conclusive to show that both variables have a significant influence on bonding performance and thus reject the null hypothesis. The inverse dependence on curing tip distance and shear bond strength was found to be steady with all types of brackets though the degree of reduction in bond strength varied based on the material composition of the bracket. The gradual decrease in the shear bond strength as the distance of curing increases is explainable by the physics of light propagation. Since the light crosses through the end tip of the curing tool and enters the separation distance, before hitting the adhesive material, the light is diverted and scattered and the irradiance decreases with the square of the distance between the light source and the surface.

This has been elucidated in studies on composite resin polymerization kinetics where lower light intensity was found to lead to low conversion rate of the monomer, high content of monomer that does not interact with other monomers and low mechanical properties of the cured material [21 , 22] The current results support those made previously that the extent of polymerization and the related hardness of the surface of resin based materials reduces considerably with increasing distance between the tip of the curing agent and the target surface [23,24] It is possible to understand the better bonding performance of stainless-steel brackets compared to polycarbonate brackets by a few reasons. The metallic bracket is welded mesh base structure, which gives a broader mechanical interlocking of the adhesive resin forming a retentive interface to resist shear displacement forces. In earlier studies, it has been regularly established that the retention of micro-mechanical nature of metal bracket bases plays a significant role in the overall bond strength, regardless of the level of adhesive polymerization [7 ,9] Also, the inflexibility of the stainless-steel bracket body assures even distribution of the applied forces throughout the bonding interface to reduce the stress concentration that could be predisposing to early bonding failure. In monocrystalline and polycrystalline forms, ceramic brackets were found to bond as well as or better than stainless-steel brackets, especially at more curing distances. This might be due to the fact that alumina based ceramic materials are inherently translucent and therefore allow a lot of curing light to pass through the bracket body to reach the layer of adhesive beneath. The transillumination capability is effective to overcome the attenuation of light with the distance of curing and ensure sufficient irradiance at adhesive-enamel interface and enhances the extent of polymerization [13,15] The similarity in the performance of monocrystalline and polycrystalline ceramic bracket as demonstrated in this study is in accordance to past reports that show that both ceramic formulations have similar bonding results even though their crystalline microstructure and optical properties are different when subjected to standardized curing environments [8,10,12] Perhaps, the most clinically important thing about this study is the much worse bonding performance of polycarbonate brackets. The thermoplastic polymer polycarbonate has intrinsic drawbacks such as a low degree of rigidity, lower elasticity modulus and lower deformation load resistance than metallic and ceramic materials. Such mechanical disadvantages can lead to flexural deformation of the base of the bracket due to shear loading that introduces an unequally distributed stress at the adhesive interface that promotes premature adhesive or cohesive failure [11]. Moreover, due to the optical opaceness of polycarbonate material, one cannot transmit light through the body of the bracket and therefore, polymerization of the adhesive under the bracket is mostly dependent on light penetrating the adhesive at the periphery of the bracket instead of transillumination. This constraint is further heightened with the increase in curing distance and a decline in the total light energy that can be utilized in polymerization. The finding that the bond strength of polycarbonate brackets at the 15 mm curing distance was less than the clinically acceptable range of 6 Mpa indicates direct clinical implications as it is possible that bond failures might be especially prone in these brackets when they are used in the posterior areas where the ideal positioning of the curing tip is challenging to attain [18]. The high level of interaction between type of bracket used and the distance of curing that the two-way ANOVA revealed only serves to remind us of the fact that the bracket materials are differently sensitive to changes in the conditions of curing. The loss in bond strength was the slowest in ceramic brackets with increasing distance of distance and supports the fact that ceramic brackets have better light transmission properties. The decline of stainless-steel brackets was moderate whereas the polycarbonate brackets exhibited the highest rate of decline and this highlights their susceptibility to poor curing conditions. The implications of these differential reactions in clinical practice include the way bracket is selected in the posterior dentition and how specific curing procedures should be applied to specific bracket materials. Results of this study are consistent with the greater mass of data on the advantages of optimization of the curing technique in the determination of consistent orthodontic bonding results. These findings have been used to make clinical advice such as keeping the curing tip as close to the tooth surface as possible, utilizing longer curing times whenever higher tip distances cannot be avoided and taking special precautions when polycarbonate brackets are used in a clinical situation where optimum light exposure is compromised [14 , 16,17]. There are a number of weaknesses of this study. Although the complex intraoral environment with salivary contamination, variation in temperature, changes in pH and masticatory loading dynamics are fully replicated in the *in vitro* design, the salivary contamination, temperature and pH changes and dynamic loading of the masticatory apparatus cannot be fully replicated in the *in vitro* design. The results of the assessment of one LED curing apparatus and adhesive system restrict the extrapolation of the results to other market products with various spectral values and resin compositions. This could be because of the lack of thermocycling or fatigue loading protocols which could have led to bond strength values which are not a true representation of long-term clinical performance. Also, the index of adhesive remnant assessment to define the patterns of failure mode was not present in this study and would have given more information on how bond failure occurs under various conditions of the experiment. The next round of studies needs to include artificial ageing regimens, test various systems of curing with different levels of output and spectral properties, the adhesive remnant index test and hopefully, prospective clinical trials may be integrated to provide laboratory findings with real intraoral conditions. Experiments that would explore the interaction effects of curing distance, exposure time and light intensity of factorial designs would further streamline clinical guidelines on how orthodontic bonding procedures are optimized.

## Conclusion:

Shear bond strength of orthodontic brackets is influenced by both material type and curing conditions. Increasing the distance of the light-curing source reduces bonding effectiveness, potentially affecting clinical performance. Optimizing curing technique and appropriate bracket selection are essential to ensure reliable and durable orthodontic bonding outcomes.

## Figures and Tables

**Table 1:** Mean shear bond strength (MPa) of different orthodontic brackets at varying LED curing tip distances

**Curing Tip Distance (mm)**	**Stainless Steel**	**Polycarbonate**	**Monocrystalline Ceramic**	**Polycrystalline Ceramic**
0	13.29 ± 1.42	9.09 ± 1.18	13.70 ± 1.35	14.24 ± 1.51
6	12.80 ± 1.38	7.20 ± 1.05	13.10 ± 1.29	13.60 ± 1.44
8	11.20 ± 1.25	6.10 ± 0.94	11.90 ± 1.21	12.30 ± 1.33
10	9.90 ± 1.16	5.30 ± 0.88	11.00 ± 1.14	11.50 ± 1.22
15	8.77 ± 1.08	4.71 ± 0.79	10.23 ± 1.06	10.62 ± 1.15
Values are expressed
as mean standard
deviation in megapascals (MPa).

**Table 2:** Two-way ANOVA results for the effects of bracket type and curing tip distance on shear bond strength

**Source of Variation**	**Sum of Squares**	**Degrees of Freedom**	**Mean Square**		**p Value**
Bracket type	512.36	3	170.79	48.72	< 0.001*
Curing distance	387.24	4	96.81	27.62	< 0.001*
Bracket type x Curing distance	58.91	12	4.91	3.14	< 0.05*
Error	631.08	180	3.51	-	-
Total	1589.59	199	-	-	-
*Statistically
significant (p< 0.05)

**Table 3:** Post-hoc pairwise comparisons (Tukey's HSD) between bracket types at selected curing distances

**Comparison**	**0 mm (p value)**	**8 mm (p value)**	**15 mm (p value)**
SS vs. Polycarbonate	< 0.001*	< 0.001*	< 0.001*
SS vs. Monocrystalline	0.842	0.412	0.038*
SS vs. Polycrystalline	0.356	0.178	0.012*
Polycarbonate vs. Monocrystalline	< 0.001*	< 0.001*	< 0.001*
Polycarbonate vs. Polycrystalline	< 0.001*	< 0.001*	< 0.001*
Monocrystalline vs. Polycrystalline	0.721	0.689	0.783
*statistically
significant (p <l 0.05)
